# Associations Between Long-Term Exposure to Air Pollutants and Prostate Cancer in a Large Taiwanese Population

**DOI:** 10.7150/ijms.109687

**Published:** 2025-05-31

**Authors:** Jiun-Hung Geng, Chia-Yang Li, Ming-Tsang Wu, Szu-Chia Chen, Shu-Pin Huang

**Affiliations:** 1 Graduate Institute of Clinical Medicine, College of Medicine, Kaohsiung Medical University, Kaohsiung 807378, Taiwan; 2 Department of Urology, Kaohsiung Municipal Siaogang Hospital, Kaohsiung 812015, Taiwan; 3 Department of Urology, Kaohsiung Medical University Hospital, Kaohsiung Medical University, Kaohsiung 807378, Taiwan; 4 Department of Urology, School of Medicine, College of Medicine, Kaohsiung Medical University 807378, Kaohsiung, Taiwan; 5 Research Center for Environmental Medicine, Kaohsiung Medical University, Kaohsiung 807378, Taiwan; 6 Graduate Institute of Medicine, College of Medicine, Kaohsiung Medical University, Kaohsiung 807378, Taiwan; 7 Ph.D. Program in Environmental and Occupational Medicine, College of Medicine, Kaohsiung Medical University, Kaohsiung 807378, Taiwan; 8 Department of Internal Medicine, Kaohsiung Municipal Siaogang Hospital, Kaohsiung Medical University, Kaohsiung 812015, Taiwan; 9 Department of Internal Medicine, Division of Nephrology, Kaohsiung Medical University Hospital, Kaohsiung Medical University, Kaohsiung 807378, Taiwan; 10 Faculty of Medicine, College of Medicine, Kaohsiung Medical University, Kaohsiung 807378, Taiwan; 11 Institute of Medical Science and Technology, College of Medicine, National Sun Yat-Sen University, Kaohsiung 804201, Taiwan

**Keywords:** Prostate cancer, Air pollution, Particulate matter, Nitrogen oxides, Sulfur dioxide, PM2.5, PM10, Ozone, Carbon monoxide, Risk factors

## Abstract

Air pollution is associated with various illnesses including cancers, of which prostate cancer is one of the most prevalent malignancies in men. Emerging evidence has suggested that air pollution is a potential risk factor for prostate cancer. This study aimed to explore the relationship between air pollution and prostate cancer in a Taiwanese population. Using data from the Kaohsiung Medical University Hospital Database, we conducted a case-control study to identify patients with prostate cancer, and matched them by age with individuals without prostate cancer. Environmental pollution indices including particulate matter (PM), nitrogen oxides (NOx), sulfur dioxide (SO2), ozone (O3) and carbon monoxide (CO) were correlated with the patients' addresses using data from the Taiwan Central Air Quality Monitoring Network. The analysis included 3541 prostate cancer patients and 7082 age-matched controls. After adjusting for confounders, conditional logistic regression analysis demonstrated significant associations of prostate cancer with PM2.5 (odds ratio [95% confidence interval]: 1.240 [1.134-1.356]) and CO (odds ratio [95% confidence interval]: 1.105 [1.025-1.192]) at the index date, with similar associations observed for average exposure levels over 1, 2, 3, and 5 years prior to the index date. Furthermore, sensitivity analyses revealed that the odds ratios for combined-risk Z-score exposure at the index date and over these same time periods were 1.029, 1.033, 1.034, 1.034, and 1.033, respectively. These findings suggest that prolonged exposure to multiple air pollutants collectively contributes to prostate cancer risk. Further investigations are needed to validate these findings and explore potential underlying mechanisms.

## Introduction

Prostate cancer (PCa) affects approximately 7,000 men in Taiwan annually 1. While treatments for metastatic PCa have advanced and subsequently increased life expectancy 2-4, nearly all patients receiving hormonal therapy ultimately progress to castration-resistant prostate cancer (CRPC), often leading to fatal outcomes. Despite the survival benefits seen with next-generation hormonal agents such as enzalutamide and abiraterone for metastatic CRPC, drug resistance remains a concern 3, 5. PCa patients experience substantial physical and emotional distress, particularly as the disease metastasizes, becoming essentially incurable. Identifying risk factors for PCa and devising preventive strategies is therefore critical.

According to the 2013 Global Disease Burden Assessment, outdoor air pollution contributes to over 3% of annual lives lost or disability 6. Pollution exerts harmful effects through oxidative stress, inflammation, and immune responses 7-10. Air pollution, a mix of gases and particles, encompasses common pollutants such as particulate matter (PM), nitrogen oxides (NOx), sulfur dioxide (SO2), ozone (O3) and carbon monoxide (CO), and it is correlated with respiratory and cardiovascular diseases, and certain cancers 11-13.

Several studies have investigated the association between exposure to air pollutants, and particulate matter with an aerodynamic diameter of 2.5 μm or less (PM2.5), and the risk of PCa 14, 15. A study conducted in Canada in 2022 linked prolonged PM2.5 exposure to an increased risk of PCa, with a 28% increase per quartile elevation in PM2.5 even after adjusting for age, race, and socioeconomic factors 15. In a study conducted in Taiwan in 2019, each 1 μg/m3 increment in PM2.5 was associated with a 13.1% increase in the incidence of cancer 16.

Although reports suggest a correlation between particulate matter with an aerodynamic diameter of 10 μm or less (PM10) and PCa, the evidence is weaker compared to PM2.5. One study suggested a positive association between PM10 and PCa, with a 23% increase in the risk of PCa per 10 μg/m^3^ elevation in PM10 17. A Danish study reported a modest 1.06 risk ratio per 10 parts per billion (ppb) nitrogen dioxide (NO2) exposure 17. However, contradictory findings exist, such as a 2016 Canadian study which did not find a link between NO2 and PCa mortality 15. Further research, especially regarding the role of nitrous oxide (NO), NO2, and NOx in the risk of PCa is therefore necessary.

Overall, evidence regarding the association between exposure to PM2.5, PM10, NO, NO2, NOx, SO2, O3 and CO with PCa is limited and inconsistent. Therefore, this study aimed to explore the correlation between air pollution and PCa among Taiwanese men using data derived from the Taiwan Central Air Quality Monitoring Network in conjunction with an advanced medical system database.

## Methods

### Database and ethics statement

This study used data from the Kaohsiung Medical University Hospital Research Database (KMUHRD), which is comprised of electronic medical record data from various healthcare facilities within the KMU health system 18. Established in 1957, this system predominantly serves the southern region of Taiwan through two regional hospitals and a medical center hospital. The KMUHRD contains comprehensive data on outpatient visits, hospital admissions, dental care, pharmaceutical records, and patient laboratory data 18. To comply with the Personal Information Protection Act and ensure confidentiality, all identifiable personal data are encrypted 18. This study strictly adhered to the ethical protocols established by the Institutional Review Board of Kaohsiung Medical University Hospital (IRB number: KMUHIRB-E(I)-20200002), which were accepted on February 11, 2020 and remain in effect until December 31, 2028, alongside the principles outlined in the Helsinki Declaration of the World Medical Association.

### Study population

**Figure 1:**
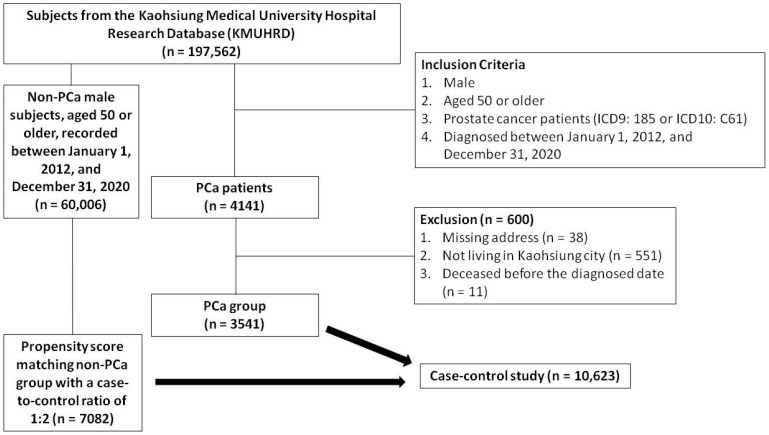
Flow chart of study enrollment.

A total of 197,562 subjects were identified in the KMUHRD. The PCa group consisted of men aged 50 years or older, diagnosed between January 1, 2012 and December 31, 2020, and identified using ICD9 diagnosis code 185 or ICD10 diagnosis code C61 in outpatient and hospitalization records. Individuals with incomplete addresses, those not living in Kaohsiung, and those who died before the diagnosis were excluded. A total of 4141 patients diagnosed with PCa were initially identified in the KMUHRD based on the inclusion criteria. After applying the exclusion criteria, 3541 patients were included as the PCa cohort. An additional cohort of 60,006 male subjects without PCa, aged 50 years or older, recorded between January 1, 2012 and December 31, 2020 were also obtained from the KMUHRD. Comprehensive clinical data were available for both groups, including demographics, comorbidities, Charlson Comorbidity Index, residence, mortality, and medication history (including dyslipidemia, hypertension, diabetes mellitus, and analgesics). Propensity score matching was conducted based on baseline characteristics using logistic regression, with each case matched with two controls at a 1:2 ratio based on propensity scores. The index date of the cases was defined as the data of PCa diagnosis, and this date was also used for the matched control pairs. The study enrollment process with details of the inclusion and exclusion criteria is shown in **Figure 1**.

### Air pollutant assessments

**Figure 2:**
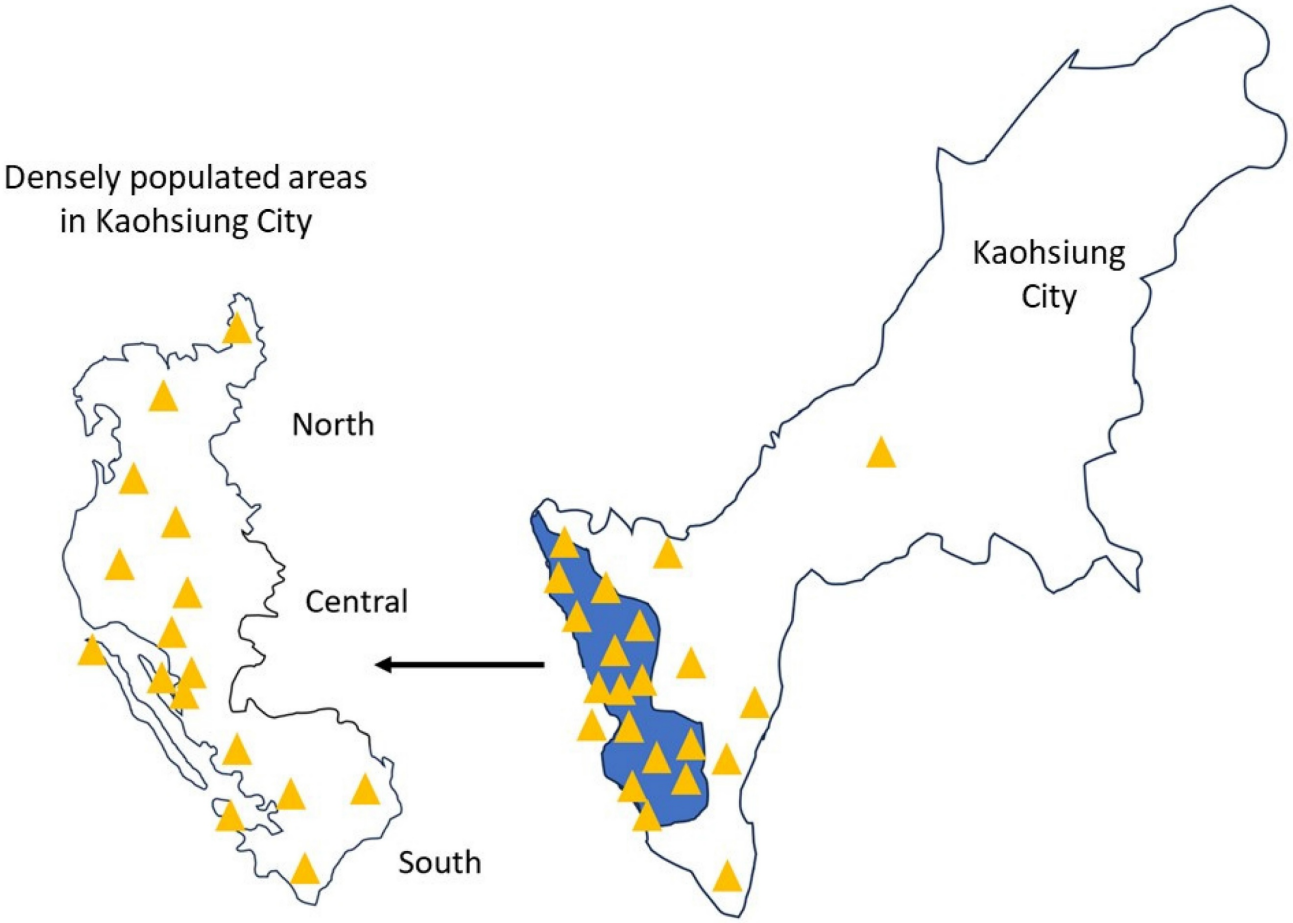
Central Air Quality Monitoring Network in Kaohsiung City.

We linked the residential addresses of the study subjects with air pollution data from the Taiwan Central Air Quality Monitoring Network, matching each address with the nearest monitoring station to retrieve relevant records 11, 19. The Taiwan Central Air Quality Monitoring Network comprises 21 general air quality monitoring stations located in densely populated areas or regions susceptible to elevated pollution levels in Kaohsiung City, divided into north, central, and south regions (**Figure 2**) 19. These stations are strategically positioned to consider factors such as pollution sources, geographical and meteorological conditions, population density, and effectiveness in evaluating pollution control measures 19. Similarly, the placement of sampling ports follows meticulous guidelines to avoid the direct influence of pollution, and ensure obstruction-free airflow and accurate pollutant concentration readings. The height of the sampling port is determined based on the vertical distribution of pollutants around the station 19. Using these data, we estimated the daily mean concentrations of PM2.5, PM10, NO, NO2, NOx, SO2, O3 and CO from 1993 to 2022 for each participant.

### PCa assessments

The diagnosis of PCa was identified based on specific patient codes (ICD9: 185 or ICD10: C61) from the Taiwan Cancer Registry. This government-led body compiles cancer data from 22,520,776 individuals, and requires cancer reports from both private and public hospitals. The extensive National Health Insurance program in Taiwan has covered cancer care since March 1995, and ensures highly accurate case records. The registry operates following standardized procedures, and it is overseen by experts and subjected to stringent computer checks and regular audits for precision and consistency, minimizing errors and discrepancies.

### Potential confounders

Besides age 20, we controlled for various established confounders associated with PCa, including geographic air pollution zone (Central, North, and South Kaohsiung) 21, benign prostate hyperplasia (ICD9: 600 or ICD10: N40) 20, and other comorbidities including cerebrovascular disease (ICD9: 430-438 or ICD10: I67) 20, hypertension (ICD9: 401.1 or ICD10: I10) 22, diabetes mellitus (ICD9: 250 or ICD10: E11) 20, dyslipidemia (ICD9: 272 or ICD10: E78) 22, congestive heart failure (ICD9: 428 or ICD10: E50) 23, chronic kidney disease (ICD9: 585 or ICD10: N18) 24, chronic obstructive pulmonary disease (ICD9: 490-496 or ICD10: J44) 25, myocardial infarction (ICD9: 410 or ICD10: I21) 23, and peripheral vascular disease (ICD9: 443.9 or ICD10: I73.9) 23. In addition, we identified medications potentially associated with PCa, including treatments for benign prostate hyperplasia (alpha blockers and 5α-reductase inhibitors) 26, dyslipidemia (statins) 22, hypertension (angiotensin-converting enzyme inhibitors, angiotensin 2 receptor blockers, calcium channel blockers, and beta blockers) 22, diabetes mellitus (SGLT2 inhibitors, biguanides) 22, aspirin 22, and nonsteroidal anti-inflammatory drugs (NSAIDs) 22. Data on these confounders were extracted from all claims made within 1 year before the index date.

### Statistical analyses

Clinical characteristics were presented as categorical data (number, percent) and compared between the PCa and non-PCa groups using Pearson's chi-square test. Air pollutant concentrations at various intervals before the index date were presented as continuous data (mean, standard deviation, minimum, maximum, and interquartile range), with differences between the PCa and non-PCa groups assessed using a two-sample test. Normality was evaluated using the Kolmogorov-Smirnov test, and for non-normally distributed continuous variables, including air pollutant levels, data transformations were applied, such as presenting values as interquartile ranges (IQRs) when distributions were skewed. Conditional logistic regression was performed to examine associations between air pollutant exposure and PCa risk. Given the presence of multiple pollutants, which could affect model validity, collinearity analysis was conducted by examining the correlation matrix in the logistic regression model and performing variance inflation factor (VIF) analysis in a linear regression model, with multicollinearity defined as |r| > 0.8 or VIF > 5; pollutants exhibiting severe multicollinearity were excluded to ensure analytical robustness. To further explore risk patterns, we standardized the concentrations of eight air pollutants (PM10, PM2.5, NO, NO2, NOx, SO2, O3, and CO) using Z-score normalization, calculated as *Z = (X - µ)/σ*, where *X* represents the raw pollutant concentration, μ is the mean, and *σ* is the standard deviation. A combined-risk Z-score, defined as the mean of the Z-scores across all pollutants, was used to represent overall air pollution exposure. For sensitivity analysis, the combined-risk Z-score was treated as a continuous variable in regression models to assess the robustness of the findings, while in the subgroup analysis, associations were examined stratified by station regions to determine whether air pollution's impact on PCa risk varied across locations. A significance level of < 0.05 was considered, and all analyses were conducted using SAS (version 9.4, Cary, North Carolina).

## Results

### Clinical characteristics of the study participants

The clinical characteristics of the PCa and non-PCa groups both before and after propensity score matching are shown in **Table 1**. Following propensity score matching, the PCa and non-PCa groups comprised 3541 and 7082 subjects, respectively. In the PCa group, 2.1% were aged 50-54 years, while the majority (42.8%) were over 75. The PCa group had high rates of comorbidities, including 45.7% with hypertension, 7.5% with diabetes mellitus, 22.9% with dyslipidemia, and 12.2% with chronic kidney disease. Notably, the usage rates of 5α-reductase inhibitors (0.7% vs 0.3%), alpha blockers (28.4% vs 6.7%), and NASIDs (10.1% vs 7.2%) were higher in the PCa group compared to the non-PCa group.

### The average levels of air pollutant exposure in all subjects

The average levels of air pollutant exposure in the study population are presented in **Supplemental Table 1**. At the index date, the average exposure levels of PM10, PM2.5, NO, NO2, NOx, SO2, O3 and CO were 60.25 ± 12.87 μg/m^3^, 29.40 ± 7.39 μg/m^3^, 4.13 ± 1.43 ppb, 18.32 ± 3.30 ppb, 22.43 ± 4.60 ppb, 4.67 ± 1.58 ppb, 28.37 ± 2.91 ppb, and 0.50 ± 0.09 parts per million (ppm), respectively. At 1 year before the index date, the average exposure levels of PM10, PM2.5, NO, NO2, NOx, and SO2 were 63.46 ± 12.78 μg/m^3^, 31.69 ± 8.65 μg/m^3^, 4.43 ± 1.62 ppb, 18.85 ± 3.35 ppb, 23.28 ± 4.82 ppb, 5.17 ± 1.80 ppb, 28.27 ± 2.91 ppb, and 0.51 ± 0.10 ppm, respectively. Similar average levels of air pollutant exposure were observed at 2 years, 3 years, and 5 years before the index date.

### The average levels of air pollutant exposure in the PCa and non-PCa groups

The average levels of air pollutant exposure in both the PCa and non-PCa groups for each of the 5 years before the index date are shown in **Table 2**. At the index date, the average exposure levels of PM10, PM2.5, NO, NO2, NOx, SO2, O3 and CO were 60.70 ± 12.80 μg/m^3^, 29.44 ± 7.31 μg/m^3^, 4.09 ± 1.39 ppb, 18.26 ± 3.25 ppb, 22.34 ± 4.51 ppb, 4.61 ± 1.51 ppb, 28.39 ± 2.90 ppb, and 0.5 0 ± 0.10 ppm in the PCa group, and 60.03 ± 12.89 μg/m^3^, 29.37 ± 7.44 μg/m3, 4.15 ± 1.45 ppb, 18.35 ± 3.33 ppb, 22.48 ± 4.64 ppb, and 4.70 ± 1.62 ppb, 28.37 ± 2.92 ppb, and 0.49 ± 0.09 ppm in the non-PCa group, respectively. Similar average exposure levels were observed at the index date, and at 1 year, 2 years, 3 years, and 5 years (**Table 2**).

Specifically, the average PM10 levels in the PCa group were 60.70 ± 12.80 μg/m^3^, 63.90 ± 12.54 μg/m^3^, 65.79 ± 11.42 μg/m^3^, 67.39 ± 10.57 μg/m^3^, and 69.36 ± 9.49 μg/m^3^ at the index date, 1 year, 2 years, 3 years, and 5 years before the index date, respectively (**Table 2**). In comparison, the average PM10 levels in the non-PCa group were 60.03 ± 12.89 μg/m^3^, 63.24 ± 12.90 μg/m^3^, 65.15 ± 11.68 μg/m^3^, 66.81 ± 10.74 μg/m^3^, and 68.89 ± 9.59 μg/m^3^ at the same respective intervals. There were significant differences in PM10, SO2, and CO levels between the PCa and non-PCa groups at all time points. However, there were no significant differences in PM2.5, NO, NO2, and NOx between the two groups before the index date (**Table 2**).

### Association between air pollutant exposure and PCa

All air pollutant levels exhibited non-normal distribution according to the Kolmogorov-Smirnov test (p-value < 0.05). To address this, we used the IQR method, and the resulting data are presented in **Supplementary Table 2**, categorized by the PCa and non-PCa groups. Then, we conducted a collinearity analysis of the air pollutant variables as shown in **Supplementary Table 3** and **Supplementary Table 4**. Our findings indicate that only PM2.5 and CO do not exhibit severe multicollinearity. Therefore, we present the associations of PM2.5 and CO with PCa risk in **Table 3**, using conditional logistic regression models adjusted for station regions and co-medications. Our results found that the odds ratios for PM2.5 exposure at the index date, as well as the 1-year, 2-year, 3-year, and 5-year average exposure levels before the index date, were 1.240, 1.359, 1.393, 1.467, and 1.413, respectively, with all p-value < 0.001. Similarly, the odds ratios for CO exposure were 1.105, 1.105, 1.105, 1.096, and 1.094, respectively, with all p-value < 0.05.

### Sensitivity and Subgroup Analyses of Air Pollutant Exposure and Prostate Cancer Risk

To further assess the impact of long-term exposure to multiple air pollutants on PCa risk prediction and explore regional variations, we conducted sensitivity and subgroup analyses. As shown in **Table 4**, the association between the combined-risk Z-score and PCa risk was evaluated, revealing that the odds ratios for exposure at the index date, as well as for 1-year, 2-year, 3-year, and 5-year average exposure levels prior to the index date, were 1.029, 1.033, 1.034, 1.034, and 1.033, respectively, with all p-values < 0.0001. Additional subgroup analyses based on station regions indicated a significant interaction effect, with the strongest association observed in central station regions (p for interaction < 0.001).

## Discussion

A total of 3,541 PCa patients and 7,082 age-matched controls were included in the present study. Our findings indicate that air pollution, particularly PM2.5 and CO, is correlated with PCa. After adjusting for confounders, we observed significant associations between prostate cancer and PM2.5 (OR [95% CI]: 1.240 [1.134-1.356]) and CO (OR [95% CI]: 1.105 [1.025-1.192]) at the index date and across multiple exposure periods. Sensitivity and subgroup analyses further confirm the impact of long-term exposure to multiple air pollutants on PCa risk prediction and explore regional variations. To the best of our knowledge, this study represents the largest case-control analysis examining the association between long-term exposure to air pollutants, especially PM2.5, CO, and PCa. Moreover, our results also provide insights into the air pollutant landscape in Taiwan, underscoring the significance of these pollutants in shaping public health policy development and strategies for disease prevention.

In this study, our findings revealed that the average yearly exposure levels of PM10, PM2.5, NO, NO2, NOx, SO2, O3 and CO at 1 year prior to the index date were 63.46 μg/m^3^, 31.69 μg/m^3^, 4.43 ppb, 18.85 ppb, 23.28 ppb, 5.17 ppb, 28.37 ppb and 0.50 ppm, respectively. The standard limits for outdoor air pollutants defined by the World Health Organization (WHO) 27, are 20 μg/m^3^ for PM10 (annual average), 10 μg/m^3^ for PM2.5 (annual average), 40 ppb for NO (annual average), 40 ppb for NO2 (annual average), 20 ppb for SO2 (24-hour average), 100 ppb for O3 (8-hour average) and 4 ppm (8-hour average). Compared to the WHO-recommended averages, the exposure levels of PM10 and PM2.5 were higher in the current study. This aligns with WHO estimates that 92% of the world's population lives in places where air quality levels exceed WHO limits 28. According to our data and the WHO, the levels of air pollutants in Taiwan are also high, underscoring the importance of understanding the effects of air pollutants on PCa.

Our main finding is that PM2.5 was a significant risk factor for PCa after adjusting for confounding variables. Importantly, we demonstrated a consistent link between PM2.5 exposure and risk of PCa, even when considering cumulative values averaged over 1, 2, 3, and 5 years before the index date. Furthermore, stratifying air pollution indicators into quartiles consistently identified PM2.5 as prominent risk factor for PCa. Supporting our findings, a study conducted in Germany also demonstrated that exposure to PM2.5 increased the risk of PCa 17, and an Italian study showed a positive correlation between long-term exposure to PM2.5 and residing in cities with elevated air pollution levels and the incidence of PCa 29. Research conducted in Denmark also revealed that men residing in regions with high air pollution levels, including PM2.5, had a greater risk of PCa compared to those in areas with lower pollution levels 30. In addition, research conducted in the Tokyo metropolitan area identified a positive association between PM2.5 exposure and PCa mortality 31. Furthermore, a study carried out in Shanghai, China, established that exposure to PM2.5 was linked to an increased risk of PCa 32. Taken together, these findings along with the present study suggest that exposure to PM2.5 may be a risk factor for the development of PCa and related mortality across diverse global populations.

We also observed that long-term CO exposure was associated with an increased risk of PCa. However, data on the relationship between CO exposure and cancer remain limited. Epidemiological studies examining populations exposed to ambient CO concentrations have generally failed to demonstrate a significant association with increased cancer risk, including PCa 39, which does not fully support our findings. This discrepancy may stem from the fact that air pollutants do not act in isolation but rather exert a collective influence on cancer development. In this context, our finding that the combined-risk Z-score of eight air pollutants is associated with an increased risk of PCa provides a possible explanation. Although evidence supports a link between air pollution and prostate cancer risk, further research is needed to specifically elucidate CO's role in PCa development. Our study contributes valuable insights into this area, particularly for investigations focusing on CO as a key pollutant.

In the present study, no significant associations were found between PM10, NO, NO2, NOx, O3 and SO2 with PCa. A review of the literature revealed that the specific relationships between PM10, NO, NO2, NOx, O3 and SO2 with PCa remain inconclusive. While some studies suggest a potential link between PM10, NO2 and SO2 exposure with an increased risk of PCa, others do not 14, 15, 17, 33-36. For example, a study conducted in Poland in 2017 33 and another in Germany in 2018 17 demonstrated a correlation between elevated PM10 levels and increased incidence of PCa. Conversely, other studies have failed to establish a clear association between PM10 and PCa 14, 34. Similar discrepancies have been reported for NO2 and SO2, with some studies indicating a positive association with the risk of PCa, and others not identifying such an association 15, 34-36. Therefore, further research is warranted to elucidate these complex relationships and to better understand the role of PM10, NO2, SO2, and other environmental factors in the risk of PCa.

In the subgroup analysis of our study, we observed a significant interaction between regions and air pollutants in relation to PCa risk. The Central region appeared to be more vulnerable, whereas the Southern and Northern regions did not show a significant association. This finding highlights the potential role of regional factors such as meteorology, pollutant composition, population characteristics, lifestyle, and healthcare access in modifying the impact of air pollution on PCa risk. Several possible explanations for these regional differences include variations in pollution levels and composition, differences in population susceptibility, and climate and atmospheric conditions (e.g., temperature, humidity, and wind patterns). Future studies should comprehensively consider these factors to disentangle the complex interplay between regional influences, potential confounders, and air pollutant exposure in PCa development.

The precise mechanisms underlying the association between PM2.5 exposure and PCa have yet to be elucidated, but they are believed to be linked to oxidative stress and inflammation 7, 9, 10. PM2.5 exposure has been demonstrated to increase reactive oxygen species production, potentially causing DNA damage and leading to mutagenesis and cancer development 37. Furthermore, PM2.5 exposure has been reported to provoke the release of inflammatory cytokines, thus fostering the proliferation and dissemination of cancer cells 38. Other potential mechanisms have also been postulated for the association between PM2.5 and risk of PCa. These mechanisms include the effect of PM2.5 exposure on disrupting hormone levels critical for PCa progression, causing alterations in gene expression via epigenetic modifications, decreasing immune function, and causing angiogenesis in the prostate gland, thereby facilitating cancer progression. The mechanism of CO.

Limited information is available on the mechanisms by which CO may contribute to the development of PCa. One potential pathway is CO's ability to bind to hemoglobin, reducing oxygen delivery to tissues and inducing hypoxia, a well-established factor in tumor progression, angiogenesis, and metastasis 39. Furthermore, CO exposure has been linked to increased oxidative stress and chronic inflammation, both of which play critical roles in carcinogenesis. Persistent inflammation may create a pro-tumorigenic microenvironment, further facilitating cancer development 39. In addition, high CO exposure is often associated with smoking and other environmental pollutants known to elevate PCa risk 39. These potential mechanisms underscore the need for further research to clarify the role of CO in PCa pathophysiology.

Several limitations to this study need to be addressed. First, while efforts were made to adjust for potential confounding variables, other factors that may influence the association between air pollution and PCa may not have been included in the analysis. Second, the study sample consisted predominantly of Asian males from Kaohsiung City, limiting the generalizability of our findings to other ethnicities or populations residing in different geographical regions. Disparities in lifestyle factors, genetic predisposition, and environmental exposure among different populations may influence the observed link between pollution and PCa. Third, air pollution estimates relied on data from the Taiwan Environmental Protection Agency's Environmental Pollution Index, which, although widely employed, may not precisely reflect individual-level exposure to air pollutants. Fourth, as with any observational study, causal inference cannot be drawn, and residual confounding from unmeasured variables may have affected the observed association between air pollution and PCa. Fifth, reliance on data from medical databases introduces potential biases related to data accuracy and completeness. Sixth, the absence of information regarding PCa severity may have impacted the interpretation of the relationship between air pollution and risk of PCa. Lastly, while this study provides evidence to support an association between air pollution and PCa, further research is needed to explore the mechanisms underlying this relationship.

## Conclusion

Our findings suggest an association between air pollution, particularly PM2.5 and CO, and PCa. These findings have potential implications for informing future policy initiatives and strategies for disease prevention. Nonetheless, further research is needed to verify these findings and elucidate the underlying mechanistic pathways involved.

## Supplementary Material

Supplementary figures and tables.

## Figures and Tables

**Table 1:** Clinical characteristics of the study participants

	Before matching			After matching		
	PCa group	Non-PCa group	p-value	PCa group	Non-PCa group	p-value
n	3541	60,006		3541	7082	
Age*						
50-54	73 (2.1%)	14,283 (23.8%)	< 0.001	73 (2.1%)	146 (2.1%)	0.162
55-64	583 (16.5%)	21,510 (35.8%)		583 (16.5%)	1166 (16.5%)	
65-74	1368 (38.6%)	15,131 (25.2%)		1368 (38.6%)	2736 (38.6%)	
75+	1517 (42.8%)	9082 (15.1%)		1517 (42.8%)	3034 (42.8%)	
Comorbidities (n, %)						
Cerebrovascular disease*	445 (12.6%)	4533 (7.6%)	< 0.001	445 (12.6%)	911 (12.9%)	0.666
Hypertension*	1618 (45.7%)	16,871 (28.1%)	< 0.001	1618 (45.7%)	3235 (45.7%)	0.989
Diabetes mellitus*	265 (7.5%)	3130 (5.2%)	< 0.001	265 (7.5%)	522 (7.4%)	0.834
Dyslipidemia*	810 (22.9%)	11,498 (19.2%)	< 0.001	810 (22.9%)	1609 (22.7%)	0.857
CHF*	208 (5.9%)	2259 (3.8%)	< 0.001	208 (5.9%)	406 (5.7%)	0.769
CKD*	433 (12.2%)	4130 (6.9%)	< 0.001	433 (12.2%)	868 (12.3%)	0.967
Myocardial infarction*	127 (3.6%)	2305 (3.8%)	0.443	127 (3.6%)	237 (3.3%)	0.521
Peripheral vascular disease	62 (1.8%)	741 (1.2%)	0.008	62 (1.8%)	130 (1.8%)	0.757
Charlson's Index Categories (n, %)*			< 0.001			0.995
0	1029 (29.1%)	32404 (54%)		1029 (29.1%)	2066 (29.2%)	
1-2	978 (27.6%)	17365 (28.9%)		978 (27.6%)	1965 (27.7%)	
3-4	504 (14.2%)	3825 (6.4%)		504 (14.2%)	997 (14.1%)	
4+	1030 (29.1%)	6412 (10.7%)				
Area			< 0.001			< 0.001
Central	2458 (69.4%)	37805 (63%)		2458 (69.4%)	4679 (66.1%)	
North	528 (14.9%)	8326 (13.9%)		528 (14.9%)	888 (12.5%)	
South	555 (15.7%)	13875 (23.1%)		555 (15.7%)	1515 (21.4%)	
Death (n,%)*			< 0.001			0.525
No	2530 (71.4%)	51993 (86.6%)		2530 (71.4%)	5018 (70.9%)	
Yes	1011 (28.6%)	8013 (13.4%)		1011 (28.6%)	2064 (29.1%)	
Medications (n, %)						
Statins				285 (8%)	569 (8%)	0.980
Antihypertension				514 (14.5%)	1031 (14.6%)	0.953
5ARIs				25 (0.7%)	19 (0.3%)	0.001
Aspirin				366 (10.3%)	758 (10.7%)	0.562
Alpha blockers				1007 (28.4%)	476 (6.7%)	< 0.001
Anti-diabetes				109 (3.1%)	269 (3.8%)	0.059
NSAIDs				357 (10.1%)	509 (7.2%)	< 0.001

* propensity score matching variable. Abbreviations: PCa: prostate cancer; CHF: congestive heart failure; CKD: chronic kidney disease; 5ARI: 5α-reductase inhibitors; NSAIDs: nonsteroidal anti-inflammatory drugs.

**Table 2:** The average levels of air pollutant exposure in the PCa and non-PCa groups

		PCa group	Non-PCa group	p-value
n		3541	7082	
Average exposure levels at the index date				
	PM10 (μg/m3)	60.70 ± 12.80	60.03 ± 12.89	0.0109
	PM2.5 (μg/m3)	29.44 ± 7.31	29.37 ± 7.44	0.6819
	NO (ppb)	4.09± 1.39	4.15 ± 1.45	0.0428
	NO2 (ppb)	18.26 ± 3.25	18.35± 3.33	0.1865
	NOx (ppb)	22.34 ± 4.51	22.48 ± 4.64	0.1238
	SO2 (ppb)	4.6 1± 1.51	4.70 ± 1.62	0.0040
	O3 (ppb)	28.39 ± 2.90	28.37 ± 2.92	0.7561
	CO (ppm)	0.50 ± 0.10	0.49 ± 0.09	0.0035
1-year average exposure levels before index date				
	PM10 (μg/m3)	63.90 ± 12.54	63.24 ± 12.90	0.0125
	PM2.5 (μg/m3)	31.77 ± 8.49	31.56 ± 8.73	0.5284
	NO (ppb)	4.41 ± 1.60	4.44 ± 1.64	0.3093
	NO2 (ppb)	18.82 ± 3.29	18.86 ± 3.38	0.5484
	NOx (ppb)	23.23 ± 4.74	23.30 ± 4.87	0.4529
	SO2 (ppb)	5.10 ± 1.72	5.20 ± 1.84	0.0100
	O3 (ppb)	28.22 ± 2.90	28.30 ± 2.91	0.2232
	CO (ppm)	0.52 ± 0.10	0.51 ± 0.10	0.0020
2-year average exposure levels before index date				
	PM10 (μg/m3)	65.79 ± 11.42	65.15 ± 11.68	0.0081
	PM2.5 (μg/m3)	32.85 ± 7.92	32.74 ± 8.13	0.5291
	NO (ppb)	4.59 ± 1.63	4.62 ± 1.67	0.3343
	NO2 (ppb)	19.26 ± 3.27	19.29 ± 3.35	0.6476
	NOx (ppb)	23.85 ± 4.78	23.91 ± 4.90	0.5334
	SO2 (ppb)	5.41 ± 1.79	5.50 ± 1.89	0.0179
	O3 (ppb)	28.10 ± 2.59	28.14 ± 2.63	0.4610
	CO (ppm)	0.52 ± 0.09	0.52 ± 0.1	0.0017
3-year average exposure levels before index date				
	PM10 (μg/m3)	67.39 ± 10.57	66.81 ± 10.74	0.0085
	PM2.5 (μg/m3)	34.00 ± 7.63	33.89 ± 7.86	0.4819
	NO (ppb)	4.75 ± 1.65	4.78 ± 1.70	0.4010
	NO2 (ppb)	19.60 ± 3.25	19.62 ± 3.31	0.7305
	NOx (ppb)	24.35 ± 4.80	24.40 ± 4.91	0.6044
	SO2 (ppb)	5.64 ± 1.79	5.72 ± 1.88	0.0202
	O3 (ppb)	27.99 ± 2.41	28.02 ± 2.44	0.5547
	CO (ppm)	0.53 ± 0.10	0.52 ± 0.10	0.0016
5-year average exposure levels before index date				
	PM10 (μg/m3)	69.36 ± 9.49	68.89 ± 9.59	0.0170
	PM2.5 (μg/m3)	35.70 ± 7.00	35.58 ± 7.21	0.4433
	NO (ppb)	5.03 ± 1.61	5.06 ± 1.67	0.3758
	NO2 (ppb)	20.07 ± 3.14	20.09 ± 3.19	0.7267
	NOx (ppb)	25.09 ± 4.66	25.14 ± 4.78	0.6096
	SO2 (ppb)	5.97 ± 1.2	6.07 ± 1.82	0.0068
	O3 (ppb)	27.85 ± 2.25	27.86 ± 2.30	0.8252
	CO (ppm)	0.54 ± 0.10	0.53 ± 0.10	0.0020

Abbreviations: PCa = prostate cancer; PM2.5 = particulate matter with an aerodynamic diameter of 2.5 μm or less; PM10 = particulate matter with an aerodynamic diameter of 10 μm or less; SO2 = sulfur dioxide; NO = nitric oxide; NO2 = nitrogen dioxide; NOx = nitrogen oxide; O3 = ozone; CO = carbon monoxide; ppb = parts per billion; ppm = parts per million.

**Table 3:** Associations of PM2.5 and CO with the risk of PCa in the present study

	Index date*			1 year*			2 years*			3 years*			5 years*		
	OR	95%CI	p-value	OR	95%CI	p-value	OR	95%CI	p-value	OR	95%CI	p-value	OR	95%CI	p-value
Station regions (ref. north)															
Central	0.780	(0.677-0.900)	0.001	0.770	(0.668-0.888)	< 0.001	0.772	(0.669-0.890)	< 0.001	0.772	(0.669-0.891)	< 0.001	0.764	(0.662-0.882)	< 0.001
South	0.580	(0.498-0.675)	< 0.001	0.564	(0.485-0.656)	< 0.001	0.564	(0.484-0.656)	< 0.001	0.561	(0.482-0.652)	< 0.001	0.551	(0.474-0.641)	< 0.001
Air pollutants (Per IQR)															
PM2.5	1.240	(1.134-1.356)	< 0.001	1.359	(1.233-1.497)	< 0.001	1.393	(1.262-1.538)	< 0.001	1.467	(1.313-1.639)	< 0.001	1.413	(1.279-1.560)	< 0.001
CO	1.105	(1.025-1.192)	0.009	1.105	(1.022-1.195)	0.012	1.105	(1.017-1.200)	0.019	1.096	(1.010-1.189)	0.028	1.094	(1.009-1.185)	0.029

* Adjusted for station regions and co-medications. Abbreviations: PCa = prostate cancer; IQR = interquartile range; OR = odds ratio; 95% CI = 95% confidence interval; PM2.5 = particulate matter with an aerodynamic diameter of 2.5 μm or less; CO = carbon monoxide.

**Table 4:** Association between each unit increase in the combined-risk Z-score for long-term pollutant exposure and prostate cancer risk

	Station regions			Central			South			North			
Air pollutants	OR*	95%CI	p-value	OR*	95%CI	p-value	OR*	95%CI	p-value	OR*	95%CI	p-value	p for interaction**
Combined risk z score													
Index date	1.029	(1.020-1.039)	< 0.0001	1.041	(1.028-1.055)	< 0.0001	0.991	(0.958-1.026)	0.616	1.008	(0.946-1.074)	0.809	0.0004
1 year	1.033	(1.024-1.043)	< 0.0001	1.045	(1.032-1.059)	< 0.0001	1.002	(0.967-1.038)	0.913	1.025	(0.955-1.101)	0.489	0.0002
2 years	1.034	(1.025-1.044)	< 0.0001	1.046	(1.033-1.059)	< 0.0001	1.000	(0.966-1.036)	0.979	1.025	(0.953-1.103)	0.501	0.0001
3 years	1.034	(1.025-1.044)	< 0.0001	1.046	(1.033-1.059)	< 0.0001	0.998	(0.964-1.033)	0.914	1.019	(0.947-1.096)	0.618	< 0.0001
5 years	1.033	(1.023-1.042)	< 0.0001	1.046	(1.032-1.060)	< 0.0001	0.994	(0.961-1.028)	0.717	1.026	(0.953-1.105)	0.491	< 0.0001

* The odds ratios are reported per unit increase in the combined-risk Z-score exposure at the index date, as well as the 1-year, 2-year, 3-year, and 5-year average exposure levels before the index date, and their association with prostate cancer risk after adjusting for covariates. ** The interaction term is the combined-risk Z-score | region, with the p-value for interaction indicating the significance of the regional differences in the effect of the combined-risk Z-score on prostate cancer risk. Abbreviations: OR = odds ratio; 95% CI = 95% confidence interval.
