# Supplementary material for: Associations Between Long-Term Exposure to Air Pollutants and Prostate Cancer in a Large Taiwanese Population
Source: Int J Med Sci. 2025 May 31;22(11):2771–81. doi: 10.7150/ijms.109687 (PMC12163608; doi:10.7150/ijms.109687)
Supplement: Supplementary file 1 — Supplementary figures and tables. [file ijmsv22p2771s1.pdf]

Associations Between Long-Term Exposure to Air Pollutants and Prostate Cancer in a Large Taiwanese Population

Supplementary Table 1. The average levels of air pollutant exposure in the present study

Average exposure levels at the index date								
	mean	Standard deviation	minimum	maximum	Q1	Q2	Q3	IQR
PM10 ($\mu g/m^3$)	60.25	12.87	32.50	82.92	50.92	61.25	70.58	19.67
PM2.5 ($\mu g/m^3$)	29.40	7.39	15.00	46.42	24.42	27.42	36.42	12.00
NO (ppb)	4.13	1.43	1.19	8.80	3.05	3.98	4.97	1.93
NO2 (ppb)	18.32	3.30	10.81	27.90	15.92	18.13	21.09	5.17
NOx (ppb)	22.43	4.60	10.88	34.30	18.95	22.26	25.54	6.59
SO2 (ppb)	4.67	1.58	2.17	9.09	3.25	4.48	6.03	2.78
O3 (ppb)	28.37	2.91	15.40	39.55	26.58	28.18	30.25	3.67
CO (ppm)	0.50	0.09	0.29	0.77	0.43	0.49	0.55	0.12
1 year average exposure levels before index date								
	mean	Standard deviation	minimum	maximum	Q1	Q2	Q3	IQR
PM10 ($\mu g/m^3$)	63.46	12.78	36.92	89.15	54.00	66.25	73.25	19.25
PM2.5 ($\mu g/m^3$)	31.69	8.65	18.50	49.58	25.25	28.33	40.17	14.92
NO (ppb)	4.43	1.62	1.19	9.13	3.21	4.17	5.33	2.12
NO2 (ppb)	18.85	3.35	10.81	27.15	16.45	18.72	21.53	5.08
NOx (ppb)	23.28	4.82	12.04	36.28	19.60	23.01	26.78	7.18
SO2 (ppb)	5.17	1.80	2.17	10.43	3.83	4.83	6.29	2.46
O3 (ppb)	28.27	2.91	15.40	36.37	26.49	28.19	30.22	3.73
CO (ppm)	0.51	0.10	0.29	0.83	0.44	0.51	0.57	0.13
2 years average exposure levels before index date								
	mean	Standard deviation	minimum	maximum	Q1	Q2	Q3	IQR
PM10 ($\mu g/m^3$)	65.36	11.59	37.96	85.67	56.42	68.58	73.00	16.58
PM2.5 ($\mu g/m^3$)	32.78	8.06	19.83	47.04	26.33	30.17	40.54	14.21
NO (ppb)	4.61	1.66	1.27	9.22	3.29	4.24	5.55	2.26
NO2 (ppb)	19.28	3.32	11.09	26.51	16.95	19.18	22.02	5.08
NOx (ppb)	23.89	4.86	12.39	35.52	20.23	23.65	27.63	7.40
SO2 (ppb)	5.47	1.86	2.24	10.72	4.05	5.04	6.53	2.48
O3 (ppb)	28.13	2.62	18.58	35.83	26.51	27.78	30.00	3.49
CO (ppm)	0.52	0.10	0.30	0.82	0.44	0.51	0.58	0.14
3 years average exposure levels before index date								
	mean	Standard deviation	minimum	maximum	Q1	Q2	Q3	IQR
PM10 ($\mu g/m^3$)	67.00	10.69	39.69	88.04	58.25	69.44	74.10	15.85
PM2.5 ($\mu g/m^3$)	33.92	7.78	21.00	48.11	26.53	33.19	41.97	15.44
NO (ppb)	4.77	1.68	1.35	9.53	3.46	4.52	5.77	2.31
NO2 (ppb)	19.61	3.29	11.24	27.06	17.23	19.54	22.35	5.12
NOx (ppb)	24.38	4.87	12.97	36.40	20.68	24.17	28.07	7.39

SO2 (ppb)	5.70	1.85	2.40	10.72	4.28	5.40	7.10	2.82
O3 (ppb)	28.01	2.43	20.86	35.13	26.44	27.70	29.44	3.00
CO (ppm)	0.52	0.10	0.31	0.84	0.45	0.52	0.59	0.14
5 years average exposure levels before index date								
	mean	Standard deviation	minimum	maximum	Q1	Q2	Q3	IQR
PM10 ($\mu g/m^3$)	69.04	9.56	40.22	88.04	62.35	70.17	74.85	12.50
PM2.5 ($\mu g/m^3$)	35.62	7.13	22.76	48.11	28.77	36.79	42.00	13.23
NO (ppb)	5.05	1.65	1.63	9.53	3.76	4.88	5.93	2.17
NO2 (ppb)	20.08	3.17	11.84	27.06	17.80	19.97	22.59	4.80
NOx (ppb)	25.13	4.74	13.75	36.40	21.48	24.88	28.58	7.11
SO2 (ppb)	6.03	1.79	2.74	10.72	4.62	5.72	7.39	2.77
O3 (ppb)	27.85	2.28	22.40	35.13	26.41	27.52	29.41	3.00
CO (ppm)	0.54	0.10	0.31	0.84	0.47	0.53	0.60	0.14

Abbreviations: PM2.5 = particle with aerodynamic diameter of 2.5 µm or less; PM10 = particle with aerodynamic diameter of 10 µm or less; SO2 = sulfur dioxide; NO = nitric oxide; NO2 = nitrogen dioxide; NOx = nitrogen oxide; O3 = ozone; CO = carbon monoxide; ppb = parts per billion; ppm = parts per million; Q1 = first quartile; Q2 = second quartile; Q3 = third quartile; IQR = interquartile range.

Supplementary Table 2. The average levels of air pollutant exposure in PCa and non-PCa groups

Variable		Total	PCa group 3541	Non-PCa group 7082	p-value
		10623	3,541 (33.3%)	7,082 (66.7%)	
At the index date					
PM10	1 - 25%	2707	858 (24.2%)	1,849 (26.1%)	0.0192
	26 - 50%	2663	900 (25.4%)	1,763 (24.9%)	.
	51 - 75%	2596	841 (23.8%)	1,755 (24.8%)	.
	76 - 100%	2657	942 (26.6%)	1,715 (24.2%)	.
PM2.5	1 - 25%	2711	889 (25.1%)	1,822 (25.7%)	0.7903
	26 - 50%	2681	892 (25.2%)	1,789 (25.3%)	.
	51 - 75%	2620	893 (25.2%)	1,727 (24.4%)	.
	76 - 100%	2611	867 (24.5%)	1,744 (24.6%)	.
NO	1 - 25%	2731	897 (25.3%)	1,834 (25.9%)	0.0013
	26 - 50%	2633	912 (25.8%)	1,721 (24.3%)	.
	51 - 75%	2612	922 (26.0%)	1,690 (23.9%)	.
	76 - 100%	2647	810 (22.9%)	1,837 (25.9%)	.
NO2	1 - 25%	2700	902 (25.5%)	1,798 (25.4%)	0.1386
	26 - 50%	2721	924 (26.1%)	1,797 (25.4%)	.
	51 - 75%	2515	866 (24.5%)	1,649 (23.3%)	.
	76 - 100%	2687	849 (24.0%)	1,838 (26.0%)	.
NOx	1 - 25%	2711	915 (25.8%)	1,796 (25.4%)	0.2336
	26 - 50%	2643	891 (25.2%)	1,752 (24.7%)	.
	51 - 75%	2636	900 (25.4%)	1,736 (24.5%)	.
	76 - 100%	2633	835 (23.6%)	1,798 (25.4%)	.
SO2	1 - 25%	2708	879 (24.8%)	1,829 (25.8%)	0.0005
	26 - 50%	2704	988 (27.9%)	1,716 (24.2%)	.
	51 - 75%	2570	840 (23.7%)	1,730 (24.4%)	.
	76 - 100%	2641	834 (23.6%)	1,807 (25.5%)	.
O3	1 - 25%	2675	889 (25.1%)	1,786 (25.2%)	0.9371
	26 - 50%	2683	884 (25.0%)	1,799 (25.4%)	.
	51 - 75%	2629	878 (24.8%)	1,751 (24.7%)	.
	76 - 100%	2636	890 (25.1%)	1,746 (24.7%)	.
CO	1 - 25%	2814	911 (25.7%)	1,903 (26.9%)	0.0073
	26 - 50%	2388	789 (22.3%)	1,599 (22.6%)	.
	51 - 75%	2854	915 (25.8%)	1,939 (27.4%)	.
	76 - 100%	2567	926 (26.2%)	1,641 (23.2%)	.
1 year before index date					

PM10	1 - 25%	2669	830 (23.4%)	1,839 (26.0%)	0.0452
	26 - 50%	2658	903 (25.5%)	1,755 (24.8%)	.
	51 - 75%	2657	908 (25.6%)	1,749 (24.7%)	.
	76 - 100%	2639	900 (25.4%)	1,739 (24.6%)	.
PM2.5	1 - 25%	2697	856 (24.2%)	1,841 (26.0%)	0.0391
	26 - 50%	2594	903 (25.5%)	1,691 (23.9%)	.
	51 - 75%	2677	923 (26.1%)	1,754 (24.8%)	.
	76 - 100%	2655	859 (24.3%)	1,796 (25.4%)	.
NO	1 - 25%	2662	862 (24.3%)	1,800 (25.4%)	0.0263
	26 - 50%	2650	926 (26.2%)	1,724 (24.3%)	.
	51 - 75%	2655	914 (25.8%)	1,741 (24.6%)	.
	76 - 100%	2656	839 (23.7%)	1,817 (25.7%)	.
NO2	1 - 25%	2661	898 (25.4%)	1,763 (24.9%)	0.0061
	26 - 50%	2697	891 (25.2%)	1,806 (25.5%)	.
	51 - 75%	2631	935 (26.4%)	1,696 (23.9%)	.
	76 - 100%	2634	817 (23.1%)	1,817 (25.7%)	.
NOx	1 - 25%	2655	896 (25.3%)	1,759 (24.8%)	0.0349
	26 - 50%	2637	867 (24.5%)	1,770 (25.0%)	.
	51 - 75%	2665	938 (26.5%)	1,727 (24.4%)	.
	76 - 100%	2666	840 (23.7%)	1,826 (25.8%)	.
SO2	1 - 25%	2648	858 (24.2%)	1,790 (25.3%)	<0.0001
	26 - 50%	2641	938 (26.5%)	1,703 (24.0%)	.
	51 - 75%	2676	947 (26.7%)	1,729 (24.4%)	.
	76 - 100%	2658	798 (22.5%)	1,860 (26.3%)	.
O3	1 - 25%	2644	888 (25.1%)	1,756 (24.8%)	0.8316
	26 - 50%	2667	873 (24.7%)	1,794 (25.3%)	.
	51 - 75%	2676	906 (25.6%)	1,770 (25.0%)	.
	76 - 100%	2636	874 (24.7%)	1,762 (24.9%)	.
CO	1 - 25%	2702	862 (24.3%)	1,840 (26.0%)	0.0213
	26 - 50%	2759	913 (25.8%)	1,846 (26.1%)	.
	51 - 75%	2523	824 (23.3%)	1,699 (24.0%)	.
	76 - 100%	2639	942 (26.6%)	1,697 (24.0%)	.
2 years before index date					
PM10	1 - 25%	2658	835 (23.6%)	1,823 (25.7%)	0.1161
	26 - 50%	2649	899 (25.4%)	1,750 (24.7%)	.
	51 - 75%	2704	916 (25.9%)	1,788 (25.2%)	.
	76 - 100%	2612	891 (25.2%)	1,721 (24.3%)	.
PM2.5	1 - 25%	2649	840 (23.7%)	1,809 (25.5%)	0.0837

	26 - 50%	2672	912 (25.8%)	1,760 (24.9%)	.
	51 - 75%	2616	909 (25.7%)	1,707 (24.1%)	.
	76 - 100%	2686	880 (24.9%)	1,806 (25.5%)	.
NO	1 - 25%	2645	862 (24.3%)	1,783 (25.2%)	0.071
	26 - 50%	2674	923 (26.1%)	1,751 (24.7%)	.
	51 - 75%	2650	914 (25.8%)	1,736 (24.5%)	.
	76 - 100%	2654	842 (23.8%)	1,812 (25.6%)	.
NO2	1 - 25%	2659	892 (25.2%)	1,767 (25.0%)	0.2372
	26 - 50%	2660	892 (25.2%)	1,768 (25.0%)	.
	51 - 75%	2631	906 (25.6%)	1,725 (24.4%)	.
	76 - 100%	2673	851 (24.0%)	1,822 (25.7%)	.
NOx	1 - 25%	2675	896 (25.3%)	1,779 (25.1%)	0.1786
	26 - 50%	2644	886 (25.0%)	1,758 (24.8%)	.
	51 - 75%	2649	916 (25.9%)	1,733 (24.5%)	.
	76 - 100%	2655	843 (23.8%)	1,812 (25.6%)	.
SO2	1 - 25%	2681	884 (25.0%)	1,797 (25.4%)	0.0016
	26 - 50%	2602	928 (26.2%)	1,674 (23.6%)	.
	51 - 75%	2720	925 (26.1%)	1,795 (25.3%)	.
	76 - 100%	2620	804 (22.7%)	1,816 (25.6%)	.
O3	1 - 25%	2658	870 (24.6%)	1,788 (25.2%)	0.2887
	26 - 50%	2707	905 (25.6%)	1,802 (25.4%)	.
	51 - 75%	2607	906 (25.6%)	1,701 (24.0%)	.
	76 - 100%	2651	860 (24.3%)	1,791 (25.3%)	.
CO	1 - 25%	2553	816 (23.0%)	1,737 (24.5%)	0.0173
	26 - 50%	2570	849 (24.0%)	1,721 (24.3%)	.
	51 - 75%	2821	918 (25.9%)	1,903 (26.9%)	.
	76 - 100%	2679	958 (27.1%)	1,721 (24.3%)	.
3 years before index date					
PM10	1 - 25%	2656	851 (24.0%)	1,805 (25.5%)	0.1216
	26 - 50%	2652	887 (25.0%)	1,765 (24.9%)	.
	51 - 75%	2640	866 (24.5%)	1,774 (25.0%)	.
	76 - 100%	2675	937 (26.5%)	1,738 (24.5%)	.
PM2.5	1 - 25%	2667	845 (23.9%)	1,822 (25.7%)	0.0065
	26 - 50%	2605	902 (25.5%)	1,703 (24.0%)	.
	51 - 75%	2698	950 (26.8%)	1,748 (24.7%)	.
	76 - 100%	2653	844 (23.8%)	1,809 (25.5%)	.
NO	1 - 25%	2620	867 (24.5%)	1,753 (24.8%)	0.1753
	26 - 50%	2676	906 (25.6%)	1,770 (25.0%)	.

	51 - 75%	2671	923 (26.1%)	1,748 (24.7%)	.
	76 - 100%	2656	845 (23.9%)	1,811 (25.6%)	.
NO2	1 - 25%	2640	885 (25.0%)	1,755 (24.8%)	0.2397
	26 - 50%	2677	905 (25.6%)	1,772 (25.0%)	.
	51 - 75%	2619	897 (25.3%)	1,722 (24.3%)	.
	76 - 100%	2687	854 (24.1%)	1,833 (25.9%)	.
NOx	1 - 25%	2652	884 (25.0%)	1,768 (25.0%)	0.2751
	26 - 50%	2682	916 (25.9%)	1,766 (24.9%)	.
	51 - 75%	2625	892 (25.2%)	1,733 (24.5%)	.
	76 - 100%	2664	849 (24.0%)	1,815 (25.6%)	.
SO2	1 - 25%	2649	877 (24.8%)	1,772 (25.0%)	0.0124
	26 - 50%	2645	940 (26.5%)	1,705 (24.1%)	.
	51 - 75%	2679	895 (25.3%)	1,784 (25.2%)	.
	76 - 100%	2650	829 (23.4%)	1,821 (25.7%)	.
O3	1 - 25%	2660	876 (24.7%)	1,784 (25.2%)	0.494
	26 - 50%	2642	859 (24.3%)	1,783 (25.2%)	.
	51 - 75%	2674	920 (26.0%)	1,754 (24.8%)	.
	76 - 100%	2647	886 (25.0%)	1,761 (24.9%)	.
CO	1 - 25%	2629	842 (23.8%)	1,787 (25.2%)	0.0143
	26 - 50%	2687	889 (25.1%)	1,798 (25.4%)	.
	51 - 75%	2579	834 (23.6%)	1,745 (24.6%)	.
	76 - 100%	2728	976 (27.6%)	1,752 (24.7%)	.
5 years before index date					
PM10	1 - 25%	2673	850 (24.0%)	1,823 (25.7%)	0.1429
	26 - 50%	2637	892 (25.2%)	1,745 (24.6%)	.
	51 - 75%	2667	881 (24.9%)	1,786 (25.2%)	.
	76 - 100%	2646	918 (25.9%)	1,728 (24.4%)	.
PM2.5	1 - 25%	2665	842 (23.8%)	1,823 (25.7%)	0.0039
	26 - 50%	2631	918 (25.9%)	1,713 (24.2%)	.
	51 - 75%	2695	946 (26.7%)	1,749 (24.7%)	.
	76 - 100%	2632	835 (23.6%)	1,797 (25.4%)	.
NO	1 - 25%	2650	859 (24.3%)	1,791 (25.3%)	0.0232
	26 - 50%	2658	924 (26.1%)	1,734 (24.5%)	.
	51 - 75%	2619	908 (25.6%)	1,711 (24.2%)	.
	76 - 100%	2696	850 (24.0%)	1,846 (26.1%)	.
NO2	1 - 25%	2656	878 (24.8%)	1,778 (25.1%)	0.0138
	26 - 50%	2632	904 (25.5%)	1,728 (24.4%)	.
	51 - 75%	2679	935 (26.4%)	1,744 (24.6%)	.

	76 - 100%	2656	824 (23.3%)	1,832 (25.9%)	.
NOx	1 - 25%	2662	873 (24.7%)	1,789 (25.3%)	0.0186
	26 - 50%	2662	931 (26.3%)	1,731 (24.4%)	.
	51 - 75%	2642	907 (25.6%)	1,735 (24.5%)	.
	76 - 100%	2657	830 (23.4%)	1,827 (25.8%)	.
SO2	1 - 25%	2674	890 (25.1%)	1,784 (25.2%)	0.0219
	26 - 50%	2642	939 (26.5%)	1,703 (24.0%)	.
	51 - 75%	2657	875 (24.7%)	1,782 (25.2%)	.
	76 - 100%	2650	837 (23.6%)	1,813 (25.6%)	.
O3	1 - 25%	2653	827 (23.4%)	1,826 (25.8%)	0.0542
	26 - 50%	2658	901 (25.4%)	1,757 (24.8%)	.
	51 - 75%	2649	911 (25.7%)	1,738 (24.5%)	.
	76 - 100%	2663	902 (25.5%)	1,761 (24.9%)	.
CO	1 - 25%	2708	875 (24.7%)	1,833 (25.9%)	0.0152
	26 - 50%	2853	943 (26.6%)	1,910 (27.0%)	.
	51 - 75%	2333	747 (21.1%)	1,586 (22.4%)	.
	76 - 100%	2729	976 (27.6%)	1,753 (24.8%)	.

Abbreviations: PCa = prostate cancer; PM2.5 = particle with aerodynamic diameter of 2.5 µm or less; PM10 = particle with aerodynamic diameter of 10 µm or less; SO2 = sulfur dioxide; NO = nitric oxide; NO2 = nitrogen dioxide; NOx = nitrogen oxide; O3 = ozone; CO = carbon monoxide; ppb = parts per billion; ppm = parts per million.

Supplementary Table 3. Risk of prostate cancer stratified by the interquartile range of air pollutant exposure and the associations between prostate cancer risk and PM10, PM2.5, NO, NO2, NOx, SO2, O3, and CO in the present study, including variance inflation factor (VIF) analysis using a linear regression model (n = 10,623).

	Index date*				1 year*				2 years*				3 years*				5 years*			
	OR	95%CI	p-value	VIF	OR	95%CI	p-value	VIF	OR	95%CI	p-value	VIF	OR	95%CI	p-value	VIF	OR	95%CI	p-value	VIF
Station regions (ref. north)																				
Central	0.772	(0.660-0.902)	0.001	2.26	0.778	(0.664-0.911)	0.002	3.25	0.771	(0.655-0.909)	0.002	3.49	0.769	(0.647-0.914)	0.003	3.84	0.764	(0.635-0.920)	0.004	4.4
South	0.649	(0.520-0.810)	0.000	2.56	0.668	(0.532-0.839)	0.001	4.49	0.630	(0.497-0.798)	0.000	4.85	0.579	(0.451-0.743)	0.000	5.48	0.533	(0.409-0.695)	<0.001	6.23
Air pollutants (Per IQR)																				
pm10	1.143	(0.994-1.314)	0.060	1.76	1.068	(0.926-1.232)	0.363	5.01	1.037	(0.898-1.198)	0.623	5.62	0.988	(0.841-1.160)	0.881	6.58	0.951	(0.816-1.108)	0.521	7.82
pm2.5	1.127	(0.985-1.290)	0.081	1.91	1.393	(1.178-1.647)	0.000	5.04	1.399	(1.175-1.666)	0.000	5.24	1.486	(1.209-1.827)	<0.001	5.79	1.466	(1.205-1.783)	<0.001	5.95
NO	1.156	(0.911-1.465)	0.233	2.18	1.043	(0.617-1.762)	0.876	81.63	0.864	(0.379-1.968)	0.728	173.98	1.062	(0.379-2.977)	0.910	289.4	0.841	(0.280-2.525)	0.757	341.97
NO2	0.764	(0.431-1.354)	0.356	10.79	0.762	(0.222-2.623)	0.667	331.93	0.453	(0.072-2.842)	0.398	679.23	0.628	(0.065-6.026)	0.687	1071.22	0.309	(0.028-3.342)	0.333	1195.77
NOx	0.828	(0.423-1.620)	0.581	12.40	1.037	(0.186-5.794)	0.967	668.25	1.993	(0.141-28.103)	0.610	1442.58	1.047	(0.042-26.356)	0.978	2302.12	2.559	(0.079-82.769)	0.596	2585.11
SO2	1.157	(0.947-1.412)	0.153	3.68	0.949	(0.768-1.174)	0.630	13.17	1.035	(0.827-1.297)	0.763	15.69	1.162	(0.879-1.536)	0.290	18.68	1.258	(0.927-1.707)	0.140	21.9
O3	1.026	(0.947-1.111)	0.528	1.33	0.978	(0.897-1.067)	0.616	2.65	0.979	(0.885-1.083)	0.681	3.32	0.945	(0.851-1.049)	0.286	4.19	0.923	(0.812-1.050)	0.222	5.59
CO	1.317	(1.139-1.522)	<0.001	1.97	1.264	(1.075-1.486)	0.005	8.32	1.300	(1.094-1.545)	0.003	8.10	1.297	(1.088-1.547)	0.004	8.53	1.325	(1.101-1.594)	0.003	9.26

* Adjusted for station regions and co-medications

Abbreviations: PM2.5 = particle with aerodynamic diameter of 2.5 µm or less; PM10 = particle with aerodynamic diameter of 10 µm or less; SO2 = sulfur dioxide; NO = nitric oxide; NO2 = nitrogen dioxide; NOx = nitrogen oxide; O3 = ozone; CO = carbon monoxide; ppb = parts per billion; ppm = parts per million; IQR = interquartile range; OR = odds ratio; 95% CI = 95% confidence interval; VIF = variance inflation factor.

Supplementary Table 4. Correlation Matrix of Air Pollutants and Multicollinearity Assessment in the Logistic Regression Model

Estimated Correlation Matrix, index year								
Air pollutants (per IQR)	pm10	pm2.5	NO	NO2	NOx	SO2	O3	CO
pm10	1	-0.6057	0.2469	-0.0423	0.0103	-0.2467	0.1934	-0.3293
pm2.5	-0.6057	1	-0.1165	0.0157	-0.0008	-0.2082	-0.3194	0.0129
NO	0.2469	-0.1165	1	0.6632	-0.822	-0.159	0.0547	-0.0337
NO2	-0.0423	0.0157	0.6632	1	-0.9247	-0.2167	-0.0226	-0.2086
NOx	0.0103	-0.0008	-0.822	-0.9247	1	0.027	0.1193	-0.006
SO2	-0.2467	-0.2082	-0.159	-0.2167	0.027	1	-0.1409	0.4568
O3	0.1934	-0.3194	0.0547	-0.0226	0.1193	-0.1409	1	0.0425
CO	-0.3293	0.0129	-0.0337	-0.2086	-0.006	0.4568	0.0425	1
Estimated Correlation Matrix, 1-year average exposure levels before index date								
Air pollutants (per IQR)	pm10	pm2.5	NO	NO2	NOx	SO2	O3	CO
pm10	1	-0.5876	0.1151	-0.0305	0.0162	-0.2426	0.1922	-0.3004
pm2.5	-0.5876	1	-0.0919	0.0409	0.0061	-0.3219	-0.308	-0.0936
NO	0.1151	-0.0919	1	0.9317	-0.9643	-0.0685	0.0277	-0.0214
NO2	-0.0305	0.0409	0.9317	1	-0.9813	-0.1097	-0.0029	-0.1075
NOx	0.0162	0.0061	-0.9643	-0.9813	1	-0.0118	0.0582	-0.0173
SO2	-0.2426	-0.3219	-0.0685	-0.1097	-0.0118	1	-0.2075	0.5716
O3	0.1922	-0.308	0.0277	-0.0029	0.0582	-0.2075	1	-0.0132

CO	-0.3004	-0.0936	-0.0214	-0.1075	-0.0173	0.5716	-0.0132	1
Estimated Correlation Matrix, 2-year average exposure levels before index date								
Air pollutants (per IQR)	pm10	pm2.5	NO	NO2	NOx	SO2	O3	CO
pm10	1	-0.6267	0.084	-0.027	0.0191	-0.2875	0.2054	-0.3235
pm2.5	-0.6267	1	-0.0937	0.0094	0.0168	-0.204	-0.3257	-0.015
NO	0.084	-0.0937	1	0.9595	-0.98	-0.057	-0.0048	-0.0133
NO2	-0.027	0.0094	0.9595	1	-0.9888	-0.087	0.0118	-0.083
NOx	0.0191	0.0168	-0.98	-0.9888	1	-0.006	0.0524	-0.0135
SO2	-0.2875	-0.204	-0.057	-0.087	-0.006	1	-0.3011	0.6002
O3	0.2054	-0.3257	-0.0048	0.0118	0.0524	-0.3011	1	-0.1096
CO	-0.3235	-0.015	-0.0133	-0.083	-0.0135	0.6002	-0.1096	1
Estimated Correlation Matrix, 3-year average exposure levels before index date								
Air pollutants (per IQR)	pm10	pm2.5	NO	NO2	NOx	SO2	O3	CO
pm10	1	-0.6697	0.1004	-0.0091	-0.0016	-0.2832	0.1871	-0.3343
pm2.5	-0.6697	1	-0.1073	-0.0029	0.0235	-0.1144	-0.3512	0.0424
NO	0.1004	-0.1073	1	0.9642	-0.9835	-0.0746	-0.0331	-0.0227
NO2	-0.0091	-0.0029	0.9642	1	-0.9898	-0.1136	0.0082	-0.0964
NOx	-0.0016	0.0235	-0.9835	-0.9898	1	0.0232	0.0669	0.0027
SO2	-0.2832	-0.1144	-0.0746	-0.1136	0.0232	1	-0.3761	0.6362
O3	0.1871	-0.3512	-0.0331	0.0082	0.0669	-0.3761	1	-0.2043

CO	-0.3343	0.0424	-0.0227	-0.0964	0.0027	0.6362	-0.2043	1
Estimated Correlation Matrix, 5-year average exposure levels before index date								
Air pollutants (per IQR)	pm10	pm2.5	NO	NO2	NOx	SO2	O3	CO
pm10	1	-0.6993	0.1753	0.0416	-0.065	-0.2633	0.1296	-0.3132
pm2.5	-0.6993	1	-0.117	0.0005	0.0201	-0.0374	-0.3448	0.1103
NO	0.1753	-0.117	1	0.9607	-0.9837	-0.1264	-0.0825	-0.0734
NO2	0.0416	0.0005	0.9607	1	-0.9881	-0.1915	-0.0156	-0.1645
NOx	-0.065	0.0201	-0.9837	-0.9881	1	0.0892	0.1088	0.0602
SO2	-0.2633	-0.0374	-0.1264	-0.1915	0.0892	1	-0.4619	0.6865
O3	0.1296	-0.3448	-0.0825	-0.0156	0.1088	-0.4619	1	-0.3122
CO	-0.3132	0.1103	-0.0734	-0.1645	0.0602	0.6865	-0.3122	1

Abbreviations: PM2.5 = particle with aerodynamic diameter of 2.5 μm or less; PM10 = particle with aerodynamic diameter of 10 μm or less; SO2 = sulfur dioxide; NO = nitric oxide; NO2 = nitrogen dioxide; NOx = nitrogen oxide; O3 = ozone; CO = carbon monoxide; IQR = interquartile range
